# Adrenomedullin-RAMP2 System Suppresses ER Stress-Induced Tubule Cell Death and Is Involved in Kidney Protection

**DOI:** 10.1371/journal.pone.0087667

**Published:** 2014-02-05

**Authors:** Ryuichi Uetake, Takayuki Sakurai, Akiko Kamiyoshi, Yuka Ichikawa-Shindo, Hisaka Kawate, Yasuhiro Iesato, Takahiro Yoshizawa, Teruhide Koyama, Lei Yang, Yuichi Toriyama, Akihiro Yamauchi, Kyoko Igarashi, Megumu Tanaka, Takashige Kuwabara, Kiyoshi Mori, Motoko Yanagita, Masashi Mukoyama, Takayuki Shindo

**Affiliations:** 1 Department of Cardiovascular Research, Shinshu University Graduate School of Medicine, Matsumoto, Japan; 2 Department of Medicine and Clinical Science, Kyoto University Graduate School of Medicine, Kyoto, Japan; 3 Medical Innovation Center, Kyoto University Graduate School of Medicine, Kyoto, Japan; 4 Department of Nephrology, Kyoto University Graduate School of Medicine, Kyoto, Japan; University of Tokushima, Japan

## Abstract

Various bioactive peptides have been implicated in the homeostasis of organs and tissues. Adrenomedullin (AM) is a peptide with various bioactivities. AM-receptor, calcitonin-receptor-like receptor (CLR) associates with one of the subtypes of the accessory proteins, RAMPs. Among the RAMP subisoforms, only RAMP2 knockout mice (−/−) reproduce the phenotype of embryonic lethality of AM−/−, illustrating the importance of the AM-RAMP2-signaling system. Although AM and RAMP2 are abundantly expressed in kidney, their function there remains largely unknown. We used genetically modified mice to assess the pathophysiological functions of the AM-RAMP2 system. RAMP2+/− mice and their wild-type littermates were used in a streptozotocin (STZ)-induced renal injury model. The effect of STZ on glomeruli did not differ between the 2 types of mice. On the other hand, damage to the proximal urinary tubules was greater in RAMP2+/−. Tubular injury in RAMP2+/− was resistant to correction of blood glucose by insulin administration. We examined the effect of STZ on human renal proximal tubule epithelial cells (RPTECs), which express glucose transporter 2 (GLUT2), the glucose transporter that specifically takes up STZ. STZ activated the endoplasmic reticulum (ER) stress sensor protein kinase RNA-like endoplasmic reticulum kinase (PERK). AM suppressed PERK activation, its downstream signaling, and CCAAT/enhancer-binding homologous protein (CHOP)-induced cell death. We confirmed that the tubular damage was caused by ER stress-induced cell death using tunicamycin (TUN), which directly evokes ER stress. In RAMP2+/− kidneys, TUN caused severe injury with enhanced ER stress. In wild-type mice, TUN-induced tubular damage was reversed by AM administration. On the other hand, in RAMP2+/−, the rescue effect of exogenous AM was lost. These results indicate that the AM-RAMP2 system suppresses ER stress-induced tubule cell death, thereby exerting a protective effect on kidney. The AM-RAMP2 system thus has the potential to serve as a therapeutic target in kidney disease.

## Introduction

Epidemiological studies have shown that chronic kidney disease (CKD) is a serious risk factor for cardiovascular disease, and that there is a high incidence of death due to cardiovascular events among CKD patients [1]. At present there is no way to stop the progression of CKD, and new and more effective approaches to the treatment of CKD are greatly needed. During the progression of CKD, damage caused by uremic toxins can degrade renal function. For example, it was recently reported that the uremic toxin indoxyl sulfate induces endoplasmic reticulum (ER) stress that can aggravate CKD, particularly renal tubule injury [2]. More interestingly, it is now recognized that chronic albuminuria, a major symptom of CKD, is a detrimental factor that promotes CKD by enhancing ER stress and injuring tubule cells [3]. ER stress occurs as a result of the accumulation of unfolded proteins, which activates the unfolded protein response (UPR) [4]. Normally, the UPR works to maintain cellular homeostasis, but when the stress is excessive it can lead to cell death [5] and to disease [6], [7]. For example, an exaggerated ER stress response contributes to kidney disease due to glomerular and tubular damage [8]. Thus, controlling ER stress could be an effective approach to breaking the vicious cycle of CKD progression.

Originally identified as a vasodilating peptide from human pheochromocytoma [9], AM is now known to be widely secreted in a number of organs and tissues, and to be involved in a variety of biological functions [10], [11], [12], [13], [14], [15], [16], [17]. In the kidney, AM is distributed in the glomerulus, renal tubules, collecting ducts, and vasculature [18], where it contributes to the regulation of renal blood flow [19] and water/Na diuresis [20] and suppresses the growth of mesangial cells [21]. Blood AM levels are elevated in patients with hypertension or congestive heart failure [22], [23] and in patients with renal failure, and AM is elevated in proportion to the severity of their disease [22]. Moreover, it was recently shown that the blood AM level is a sensitive marker, predictive of the long-term prognosis in CKD [24].

We previously showed that homozygotic AM knockout (KO) (AM*−/−*) mice die mid-gestation due to edema and hemorrhage [25]. Heterozygotic AM KO (AM+/−) mice grow to adulthood with no apparent deficits, but show accelerated damage to some organs, including the kidneys [26]. The clear importance of endogenous AM for cardiovascular and renal function suggests a therapeutic benefit may be gained from administration of the exogenous peptide. However, AM has a very short half-life in the bloodstream [27], which limits its utility for the treatment of chronic diseases requiring long-term therapy. For that reason, we have been focusing on the AM receptor system. AM is a member of the calcitonin superfamily and acts via a G protein-coupled seven transmembrane domain receptor (GPCR), calcitonin-receptor-like receptor (CLR) [28], [29]. The specificity of CLR for its ligands is regulated by three receptor-activity-modifying proteins, RAMP1, RAMP2, and RAMP3. We previously showed that homozygotic RAMP2 KO (RAMP2*−/−*) mice die in utero and that they die from vascular abnormalities similar to those observed in AM−/− mice [30], which suggests RAMP2 is important in the cellular function of AM. In addition, we recently generated a line of endothelial cell-specific RAMP2 KO (E-RAMP2−/−) mice. Most E-RAMP2−/− mice die perinatally, and in the surviving adults we observed the spontaneous occurrence of several renal abnormalities, including polycystic kidney, hydronephrosis, and glomerular sclerosis [31]. These findings suggest that the vascular endothelial AM-RAMP2 system is involved in mediating renal homeostasis. Notably, RAMP2 is also distributed in other cell types throughout the kidney, though its significance there remains unknown.

Our aim in the present study was to further clarify the function of the AM-RAMP2 system in the kidney. For that purpose, we used adult heterozygous RAMP2 KO (RAMP2+/−) mice in a kidney injury model. Our findings indicate that the AM-RAMP2 system is a crucial regulator of ER stress in renal tubules.

## Materials and Methods

### Animals

Wild-type (WT) C57BL/6J mice were purchased from Charles River Laboratories Japan (Yokohama, Japan). RAMP2 KO mice were originally generated by our group [30]. Because homozygous RAMP2 KO mice die in utero, we used heterozygous RAMP2 KO male mice (RAMP2+/−) and their wild-type littermate male mice in this study. All animal handling procedures were performed in accordance with a protocol approved by the Ethics Committee of Shinshu University.

### Streptozotocin (STZ) Administration Model

Four-week-old mice received a single intraperitoneal injection of 250 mg/kg body weight STZ (WAKO) dissolved in 0.9% NaCl [32]. Blood glucose levels were measured at 10∶00 a.m. without fasting. Two, 4, or 12 weeks later, the mice were sacrificed and their kidneys were collected.

### Tunicamycin Administration Model

Eight-week-old mice received a single intraperitoneal injection of 0.5–1.0 mg/kg body weight tunicamycin (TUN) (Sigma). TUN was initially dissolved in dimethyl sulfoxide (DMSO), which was then diluted in 150 mM dextrose solution for injection. Four days after the TUN injection, the mice were sacrificed and their kidneys were collected [33].

### Adrenomedullin (AM) Administration Model

The AM-treated group received continuous subcutaneous administration of recombinant human AM dissolved in 0.9% NaCl at a delivery rate of 0.05 µg/kg/min using osmotic pumps (Alzet Model 2002). AM administration began 1 week before the TUN administration and continued to the end of the study.

### Cisplatin Administration Model

Eight-week-old mice received a single intraperitoneal injection of 20 mg/kg body weight cisplatin (Sigma). Three days after the cisplatin injection, the mice were sacrificed and their kidneys were collected [34].

### Cell Culture

Normal human renal proximal tubule epithelial cells (RPTECs) were purchased from Lonza. They were cultured in REGM medium (Lonza). Cells passaged up to three times were used for experimentation.

### Histology

Tissues were fixed overnight in 4% paraformaldehyde, embedded in paraffin, and cut into 1.5-µm-thick sections for histological examination. The specimens were then deparaffinized for hematoxylin/eosin (H & E) staining, periodic acid-Schiff (PAS) staining, terminal deoxynucleotidyl transferase dUTP nick end labeling (TUNEL), and immunohistochemistry using anti-LC3 antibody (Cell Signaling Technology), and nuclear staining with DAPI (Life Technologies).

### Transmission Electron Microscopy

Specimens were fixed in 2.5% glutaraldehyde (pH 7.2), embedded in epoxy resin (Epok) 812 (Oken Shoji Co.), cut into ultrathin sections, double-stained with uranyl acetate and lead citrate, and examined with an electron microscope.

### RNA Extraction and Quantitative Real-Time RT-PCR Analysis

Total RNA was extracted from tissues using Trireagent (Molecular Research Center, Inc.), after which the RNA was treated with DNA-free (Ambion) to remove contaminating DNA and then subjected to reverse transcription using a High Capacity cDNA Reverse Transcription Kit (Applied Biosystems). Quantitative real-time RT-PCR was carried out using an Applied Biosystems 7300 real-time PCR System with SYBR green (Toyobo, Japan) or Realtime PCR Master Mix (Toyobo) and TaqMan probes (MBL). The primers and probes used are listed in Table 1. Values were normalized to mouse GAPDH (Pre-Developed TaqMan assay reagents, Applied Biosystems).

**Table 1:** Primers and probes used for quantitative real-time RT-PCR.

AM Forward	CTACCGCCAGAGCATGAACC
AM Reverse	GAAATGTGCAGGTCCCGAA
AM Probe	CCCGCAGCAATGGATGCCG
CLR Forward	AGGCGTTTACCTGCACACACT
CLR Reverse	CAGGAAGCAGAGGAAACCCC
CLR Probe	ATCGTGGTGGCTGTGTTTGCGGAG
RAMP2 Forward	GCAGCCCACCTTCTCTGATC
RAMP2 Reverse	AACGGGATGAGGCAGATGG
RAMP2 Probe	CCCAGAGGATGTGCTCCTGGCCAT
RAMP3 Forward	TGCAACGAGACAGGGATGC
RAMP3 Reverse	GCATCATGTCAGCGAAGGC
RAMP3 Probe	AGAGGCTGCCTCGCTGTGGGAA
Bax Forward	AGACACCTGAGCTGACCTTGGA
Bax Reverse	GAGACACTCGCTCAGCTTCTTG
Bcl-2 Forward	TGTGAGGACCCAATCTGGAAA
Bcl-2 Reverse	TTGCAATGAATCGGGAGTTG
BiP Forward	TCTGCCATGGTTCTCACTAAAATG
BiP Reverse	CAGTAACAATTGCATGGGTAACCT
CHOP Forward	TCCAGAAGGAAGTGCATCTTCA
CHOP Reverse	GGACTCAGCTGCCATGACT
GLUT-2 Forward	AGTACAGATGAACTGCCCACAATC
GLUT-2 Reverse	ATGGCCAGCTGATGAAAAGTG
p53 Forward	TGCATGGACGATCTGTTGCT
p53 Reverse	TGACACTCGGAGGGCTTCA
TNF-α Forward	ACGGCATGGATCTCAAAGAC
TNF-α Reverse	AGATAGCAAATCGGCTGACG
MCP-1 Forward	GCAGTTAACGCCCCACTCA
MCP-1 Reverse	CCTACTCATTGGGATCATCTTGCT
p47phox Forward	ATCCTATCTGGAGCCCCTTGA
p47phox Reverse	CACCTGCGTAGTTGGGATCC
p67phox Forward	CAGACCCAAAACCCCAGAAA
p67phox Reverse	AAAGCCAAACAATACGCGGT

### Western Blot Analysis

Western blot analysis was carried out using protein extracts from RPTECs. The lysates were subjected to electrophoresis using TGX gel (Bio-Rad Laboratories), transferred to PVDF membranes (Bio-Rad Laboratories) and probed with anti-BiP, anti-ATF6, anti-PERK, anti-phospho-PERK (P-PERK), anti-IRE1, anti-phospho-IRE1 (P-IRE1), anti-eIF2α, anti-phospho-eIF2α (P- eIF2α), anti-ATF4, anti-CHOP (anti-ATF4 obtained from abcam, all others from Cell Signaling Technology), and anti-β-tubulin (Santa Cruz Biotechnology) antibodies. The blots were then developed using an ImageQuant LAS 4000 (GE Healthcare).

### Statistical Analysis

Values are expressed as the mean ± SE. Student’s t test was used to determine significant differences between two groups. One-way ANOVA followed by Fisher’s PLSD was used to determine significant differences between three groups. Pearson’s correlation coefficient was used to evaluate the correlation between two groups. Values of p<0.05 were considered significant.

## Results

### RAMP2+/− Mice Showed Enhanced Renal Tubular Injury in a STZ Nephropathy Model

**Figure 1:**
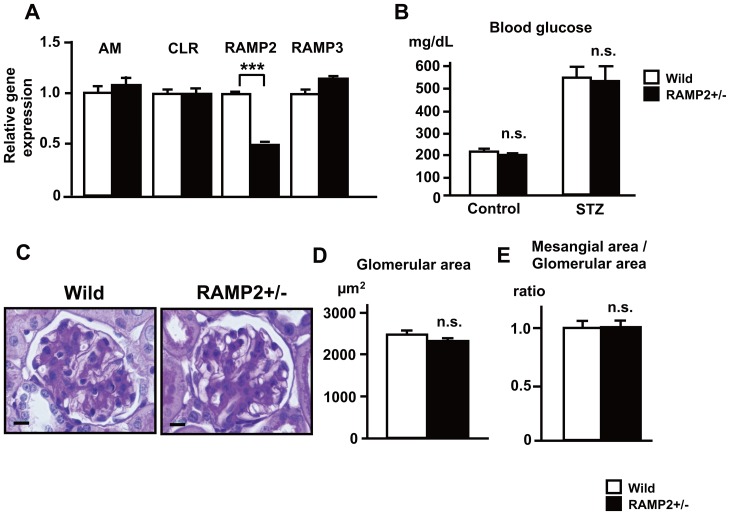
Blood glucose and glomerular changes in the STZ-induced diabetic nephropathy model. (A) Bar graphs showing relative gene expression determined by quantitative real-time PCR. Gene expression of RAMP2 was downregulated in RAMP2+/− kidney. (B) Effect of STZ treatment on blood glucose levels. In both the control and STZ groups, blood glucose levels did not differ between wild-type and RAMP2+/− mice 2 weeks after STZ administration. Data were obtained at 10∶00 a.m. without fasting. (C-E) Comparison of glomerular lesions in wild-type and RAMP2+/− kidneys following STZ treatment. (C) PAS staining of glomeruli 12 weeks after STZ administration. Scale bars = 10 µm. (D) Glomerular area. (E) Mesangial area/glomerular area ratios. n = 5 in each group. Bars are means ± SEM. ***p<0.001. n.s. = not significant.

Because RAMP2−/− mice exhibit lethal phenotypes similar to those of AM−/− mice, we hypothesized that endogenous RAMP2 is the most important in the function of AM. Heterozygous RAMP2 KO (RAMP2+/−) mice were apparently normal and fertile, though the level of RAMP2 expression in various organs, including kidney, was about half that seen in wild-type (WT) mice (Fig. 1A). To evaluate their susceptibility to renal injury, we examined RAMP2+/− mice and their WT littermates in a STZ-induced diabetic nephropathy model. Using STZ to induce hyperglycemia is a well-established method for generating experimental glomerular sclerosis. According to previous studies and the manufacturer’s information, we performed a 100 mg/kg day STZ injection for 3 consecutive days or a single injection of 250 mg/kg. Because both showed similar results for the glucose level and pathological changes, we decided to use the latter protocol thereafter.

After administering 250 mg/kg STZ to mice, hyperglycemia was observed within about 1 week. In both the control and STZ-treated groups, blood glucose levels did not differ between the WT and RAMP2+/− mice at 2 weeks (Fig. 1B). Blood pressure (BP) measured using tail cuff sphygmomanometry was not different between the 2 groups (Systolic BP: WT: 105.8±5.1, RAMP2+/−: 110.7±6.8 mmHg). After 12 weeks of STZ administration, glomeruli in both mice showed slight sclerotic changes, however, measurements of glomerular and mesangial area revealed no difference between WT and RAMP2+/− (Fig. 1C–E).

**Figure 2:**
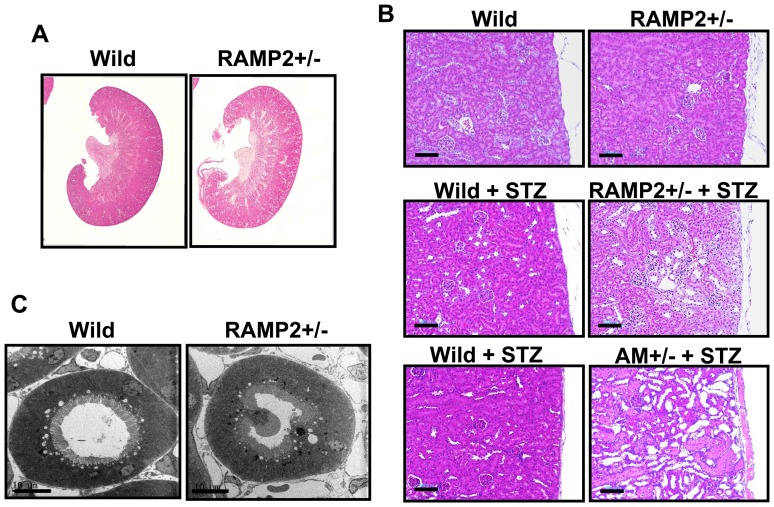
Renal tubular changes induced by the STZ treatment. (A) Cross-sections of kidneys collected from wild-type and RAMP2+/− mice 4 weeks after STZ administration (H & E staining). (B) Higher magnification of the renal cortex. STZ-administered RAMP2+/− and AM+/− showed substantial degeneration of tubule cells, which was not detected in RAMP2+/− without STZ administration. (C) Electron micrograph of a section of proximal tubule shows destruction of the brush border (protrusion of a tubule cellular membrane into the lumen) in a RAMP2+/− kidney. Scale bars = 100 µm (B), 10 µm (C).

**Figure 3:**
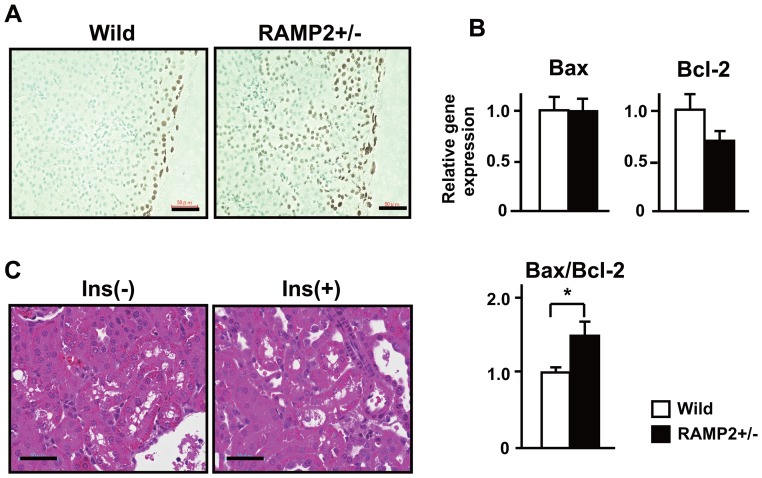
STZ-induced tubule cell death was enhanced in RAMP2+/− kidneys. (A) TUNEL assays of the renal cortex 4 weeks after STZ administration showed tubule cell death to be increased in RAMP2+/− kidneys. (B) Quantitative real-time PCR analysis of samples from the renal cortex. Shown are the relative gene expression levels of Bax and Bcl-2 (upper panels). Data from the wild-type control group were assigned a value of 1. Note the downregulation of Bcl-2 in RAMP2+/− kidneys indicated by the higher Bax/Bcl-2 ratio (lower panel). n = 5 in each group. Bars are means ± SEM. *p<0.05. (C) H&E staining of the renal cortex from RAMP2+/− kidneys 2 weeks after STZ administration, with or without insulin (Ins) treatment. Although insulin-treated RAMP2+/− mice had normal blood glucose levels, they showed the same level of tubular injury as mice not receiving insulin. Arrows indicate vacuolated proximal tubules. Scale bars = 50 µm (A), (C).

In contrast, we found urinary tubular changes to be much more prominent in STZ-treated RAMP2+/− than in WT mice. After 4 weeks of STZ administration, histological analysis showed RAMP2+/− kidneys stained poorly with H&E, suggesting interstitial edema (Fig. 2A), and in the cortical region of RAMP2+/− kidneys there was substantial degeneration of tubule cells (Fig. 2B). Some tubules were atrophic and even disappeared. No obvious inflammation, deposits, or vascular changes were found. Similar tubular damage was also confirmed in AM+/− with STZ administration, however, it was not found in RAMP2+/− without STZ administration. Using electron microscopy, the cellular membranes of tubule cells could be seen protruding into the tubule lumen in RAMP2+/− kidneys, suggesting destruction of the brush border (Fig. 2C). Taken together, these changes indicate that the proximal renal tubules of RAMP2+/− are severely damaged after STZ administration. Consistent with this finding, TUNEL assays confirmed tubule cell death to be enhanced in STZ-treated RAMP2+/− kidneys (Fig. 3A), and there was a corresponding downregulation of the anti-apoptotic factor Bcl-2 (Fig. 3B). To determine whether the tubular damage was caused by hyperglycemia, we administered insulin (glargine at a dose of 0.5–2 U daily) and STZ for 2 weeks, which normalized their blood glucose levels but did not mitigate the damage seen in RAMP2+/− kidneys (Fig. 3C). Based on these findings, we speculated that STZ acts directly to cause tubular injury, and RAMP2+/− mice were more susceptible to injury than WT mice.

### Direct Renal Tubule Cell Damage Caused by STZ

**Figure 4:**
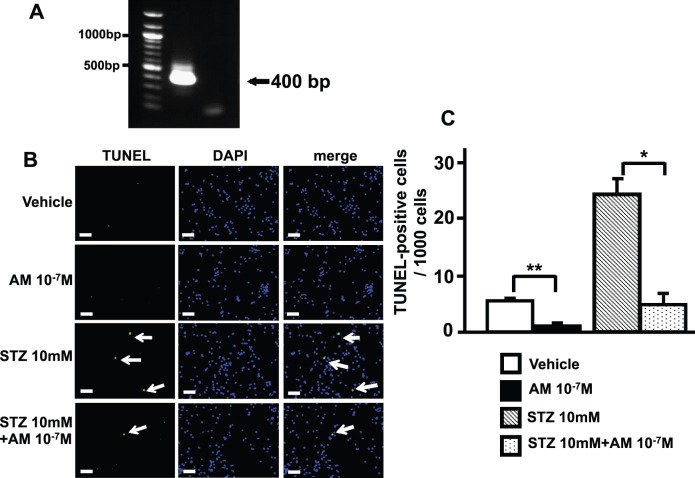
AM suppresses STZ-induced apoptosis among human renal tubule cells (RPTECs). (A) RT-PCR analysis showing the expression of GLUT2 in RPTECs. The arrow indicates the GLUT2 PCR product. (B) TUNEL assays of cultured RPTECs treated with vehicle, AM, STZ, or STZ+AM. Arrows show the TUNEL-positive cells. Scale bars = 100 µm. (C) Numbers of TUNEL-positive cells per 1,000 cultured cells. The experiments were repeated 3 times. Bars are means ± SEM. *p<0.05, **p<0.01.

We next utilized cultured human renal tubule cells (RPTECs) to evaluate the direct toxicity of STZ. STZ is a naturally occurring glucosamine-nitrosourea, and is transported into cells by the glucose transporter 2 (GLUT2) [35], which is expressed in pancreatic β cells. Notably, we found that GLUT2 is also highly expressed in renal tubule cells (Fig. 4A). When we treated RPTECs for 4 h with STZ (10 mM) or with STZ (10 mM)+AM (0.1 µM), we found that STZ induced a 4.5-fold increase in cell death, but that AM strongly inhibited the effect (Fig. 4B, C).

### STZ Induces ER Stress in Tubule Cells and the Effect is Suppressed by AM

**Figure 5:**
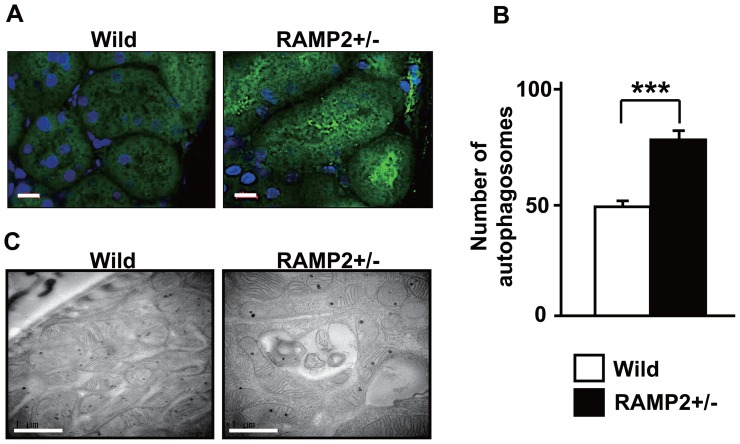
STZ enhances tubule cell autophagy in RAMP2+/− kidneys. (A) Fluorescent immunostaining of LC3 (green) and DAPI (blue) in proximal renal tubules 4 weeks after STZ administration to wild-type and RAMP2+/− mice. (B) Comparison of autophagosome numbers between wild-type and RAMP2+/− kidneys. Numbers of LC3-positve particles per microscope field were counted in LC3 immunostained sections. A total of 10 fields were analyzed. (C) Electron micrograph showing autophagosomes in a renal tubule cell. Bars are means ± SEM. ***p<0.001. Scale bars = 10 µm (A), 1 µm (C).

We also found that the number of particles immunostaining positively for LC3, an autophagosome membrane protein, was greater in STZ-treated RAMP2+/− than in WT kidneys (Fig. 5A, B). Electron microscopic observation confirmed that autophagy was enhanced in STZ-treated tubule cells (Fig. 5C), which suggests that accumulation of foreign matter within tubule cells is a key contributor to STZ-induced tubule cell death.

**Figure 6:**
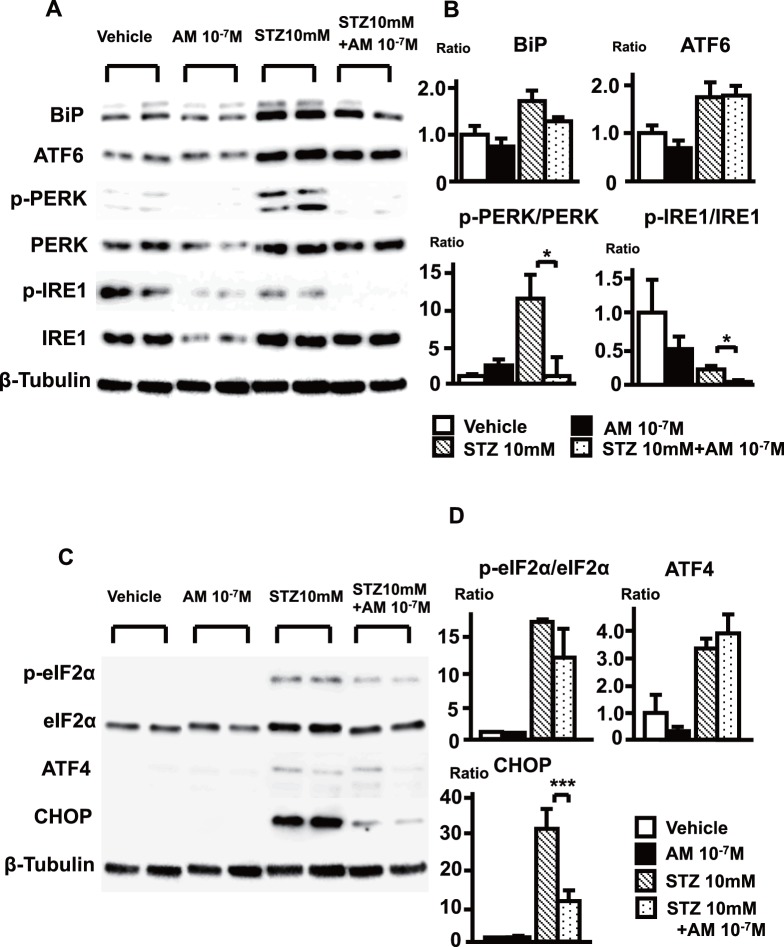
Western blot analysis of ER stress-related factors in STZ-treated RPTECs. (A) Western blot analysis of ER stress sensor molecules in STZ-treated RPTECs. (B) Optical densities of Western blots were quantified, and the relative expression of BiP and ATF6 and the p-PERK/PERK and p-IRE1/IRE1 ratios (PERK and IRE1 activation level) are shown. (C) Western blot analysis of the mediators downstream of PERK. (D) Quantification of the results in (C). The experiments were repeated 3 times. Data from the vehicle group were assigned a value of 1. Bars are means ± SEM. *p<0.05, ***p<0.001.

In that context, we examined endoplasmic reticulum (ER) stress and the unfolded protein response (UPR) system, a mechanism by which foreign materials are eliminated from cells [36]. Activating transcription factor 6 (ATF6), protein kinase RNA-like endoplasmic reticulum kinase (PERK), and inositol-requiring 1 (IRE1) are three ER stress sensing proteins, whose activations are regulated by binding immunoglobulin protein (BiP). Using Western blot analysis of cultured RPTECs, we found that STZ administration directly evoked elevation of BiP and activation of PERK (elevation of phosphorylated PERK (p-PERK)), and that AM suppressed the STZ-induced elevations of BiP and p-PERK (Fig. 6A, B). We then analyzed three downstream mediators of PERK, eukaryotic initiation factor 2α (eIF2α) ATF4, and CCAAT/enhancer-binding homologous protein (CHOP), which is a critical component of the ER stress-UPR system and induces cell death [37], [38]. Under basal conditions, little or no CHOP was detected in cultured RPTECs, but its expression was markedly upregulated by STZ, and this effect was significantly inhibited by AM (Fig. 6C, D). These results clearly show that STZ directly induces ER stress in renal tubule cells, and that AM suppresses the STZ-induced ER stress-UPR system and cell death.

### ER Stress Causes Tubular Injury and Renal Dysfunction in RAMP2+/−

**Figure 7:**
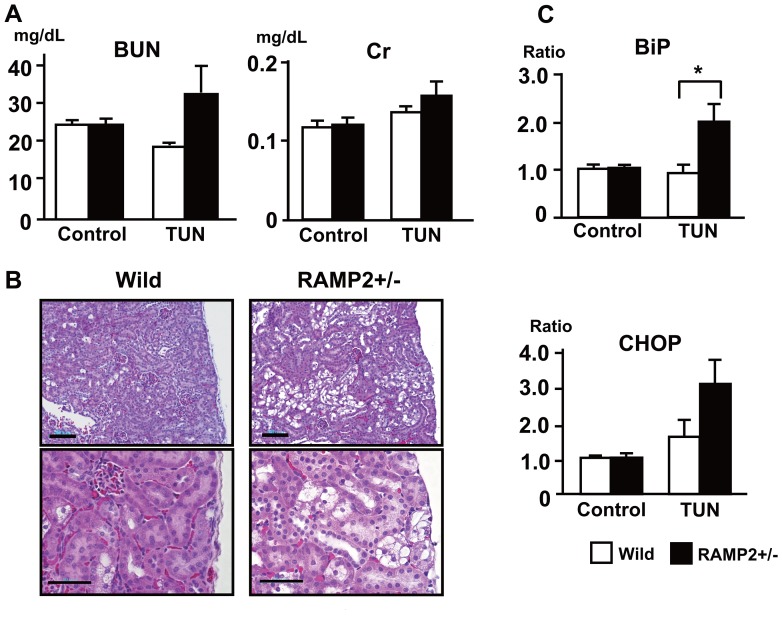
Tunicamycin (TUN) induces tubular injury and upregulation of ER stress-related factors in RAMP2+/− kidneys. (A) Serum BUN and creatinine (Cr) in wild-type and RAMP2+/−4 days after administration of vehicle (Control) or TUN (0.5 mg/kg). (B) H&E staining of the renal cortex in TUN-treated wild-type and RAMP2+/− kidneys. Scale bars in upper panels = 100 µm, lower panels = 50 µm. Sections from RAMP2+/− kidneys showed severe tubular injury with cell vacuolation. (C) Quantitative real-time PCR analysis of BiP and CHOP gene expression. TUN-treated RAMP2+/− kidneys showed upregulation of ER stress-related factors. Data from the wild-type control group were assigned a value of 1. n = 5 in each group. Bars are means ± SEM. *p<0.05.

Given that AM reduces STZ-induced ER stress in RPTECs and tubule cell damage is enhanced in the STZ-treated RAMP2+/−, we hypothesized that the endogenous AM-RAMP2 system is a key regulator controlling ER stress in tubule cells *in vivo*. To test this hypothesis, we compared the effects of administering tunicamycin (TUN) to RAMP2+/− and WT mice. TUN is known to directly induce ER stress by inhibiting the synthesis of N-linked glycoproteins (N-glycans) [39] and causing cell cycle arrest at G1 phase [36]. RAMP2+/− mice administered TUN (0.5 mg/kg) exhibited slightly elevated levels of serum BUN and Cr (Fig. 7A). RAMP2+/− showed vacuolation of tubule cells (Fig. 7B) as well as upregulation of the ER stress-related factors BiP and CHOP (Fig. 7C). In contrast, TUN had little effect on WT kidneys, suggesting endogenous AM-RAMP2 signaling exerts a protective effect.

### AM Reverses ER Stress-induced Tubular Injury

**Figure 8:**
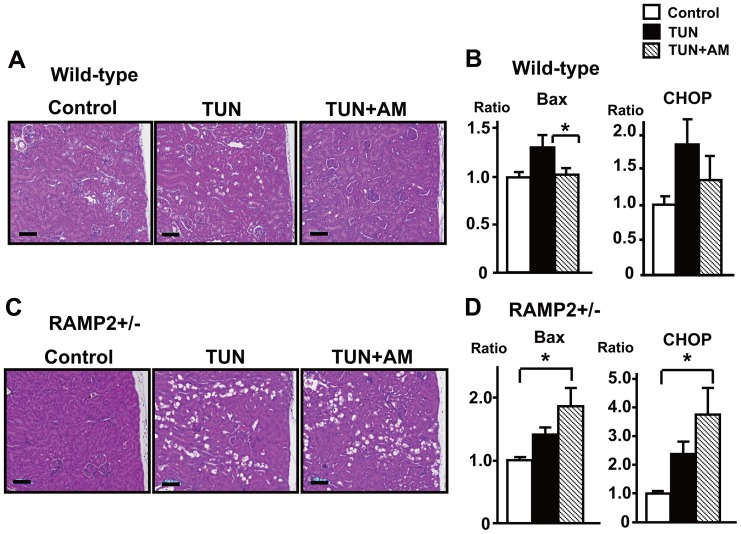
Exogenous AM reverses ER stress-induced tubular injury. (A) H&E staining of the renal cortex from wild-type mice treated with vehicle (Control), TUN, or TUN+AM. Samples were collected 4 days after TUN (1 mg/kg) administration. In the TUN+AM group, continuous AM administration at a rate of 0.05 µg/kg/min, beginning 1 week prior to TUN administration, reversed the TUN-induced tubular injury. (B) Quantitative real-time PCR analysis showing TUN-induced upregulation of ER stress-related factors CHOP and the apoptosis-related factor Bax, and their suppression by AM. (C, D) AM-treatment in RAMP2+/−. Even with the AM-treatment, RAMP2+/− showed enhanced tubular damage and upregulation of CHOP and Bax. (A, C) Scale bars = 100 µm. (B, D) Data from the control group were assigned a value of 1. n = 5 in each group. Bars are means ± SEM. *p<0.05.

We next assessed the therapeutic potential of exogenous AM administration *in vivo*. Using an implanted osmotic pump, recombinant AM was continuously administered to mice beginning 1 week prior to TUN administration. At 0.05 µg/kg/min, AM administration had no significant effect on blood pressure during the study (data not shown). In wild-type mice, a higher dose of TUN (1 mg/kg) induced renal tubular injury, and it was reduced in the AM-treated group (Fig. 8A). In addition, AM administration inhibited the renal expression of the Bax and CHOP (Fig. 8B).

On the other hand, in RAMP2+/−, the rescue effect of exogenous AM was lost; we could not detect the beneficial effects of AM, which was observed in wild-type mice (Fig. 8C, D). These results clearly indicate that the AM-RAMP2 system is important in the renal protection.

### Cisplatin Induces Enhanced Tubular Injury in RAMP2+/− with Elevation of Inflammatory and Oxidative Stresses

As TUN-induced renal injury is a rather specific model, we finally evaluated the beneficial effect of the AM-RAMP2 system using a cisplatin-induced renal injury model. Cisplatin is a widely used chemotherapeutic agent. However, it is widely known to cause renal failure as a side effect. Cisplatin selectively damages proximal tubules due to its preferential accumulation within the proximal tubule cells [40]. In cisplatin-mediated nephrotoxicity, DNA injury, decreased protein synthesis, oxidative stress, and inflammation are thought to be involved [41], [42]. Furthermore, cisplatin is reported to evoke ER stress and cell death [43].

**Figure 9:**
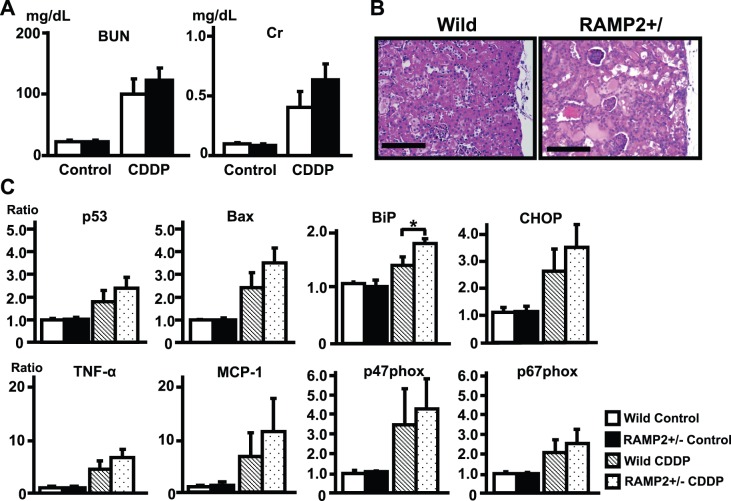
Cisplatin induces enhanced renal injury in RAMP2+/−. (A) Serum BUN and creatinine (Cr) in wild-type and RAMP2+/−3 days after administration of vehicle (Control) or cisplatin (CDDP) (20 mg/kg). (B) H&E staining of the renal cortex in CDDP-treated wild-type and RAMP2+/− kidneys. Scale bars = 100 µm. Sections from RAMP2+/− kidneys showed severe tubular injury. (C) Quantitative real-time PCR analysis of the indicated gene expression. Data from the wild-type control group were assigned a value of 1. n = 5 in each group. Bars are means ± SEM. *p<0.05.

Cisplatin administration (20 mg/kg) caused renal dysfunction with elevation of BUN and Cr, and the increases were slightly higher in RAMP2+/− (Fig. 9A). Cisplatin-administered RAMP2+/− showed enhanced tubular damage compared with wild-type mice (Fig. 9B). Cisplatin administration results in upregulation of cell death markers (p53 and Bax) and ER-stress markers (BiP and CHOP), as well as inflammatory markers (TNF-α and MCP-1) and oxidative stress markers (NADPH subunit, p47phox and p67phox), and again all were slightly higher in RAMP2+/−.

## Discussion

It has been suggested that endogenous AM makes an important contribution to the maintenance of proper renal physiological function [19], [20], [21]. It has also been reported that AM levels are elevated in renal failure [22] and are a good predictor of who will or will not experience CKD progression [24]. Consistent with that finding, the expressions of CLR and RAMPs reportedly increase with progression of renal fibrosis in experimental models of CKD [44], [45]. These reports thus implicate AM and its receptor in the development of CKD, although their precise pathophysiological significance remains unclear. In the present study, we used RAMP2 KO mice to show that endogenous AM-RAMP2 signaling exerts a protective effect on the kidney by regulating the ER stress-UPR system in the proximal renal tubules.

AM-induced effects that may work toward organ protection include the reduction of blood pressure and suppression of inflammation, oxidative stress, and fibrosis. Although we and others have shown that AM+/− mice grow to adulthood with no apparent deficits, these animals do exhibit accelerated organ damage when put under stress [13], [25], [46]. For example, we previously observed that ischemia/reperfusion injury is exacerbated in AM+/− kidneys [26]. Meanwhile, AM-overexpressing transgenic mice showed resistance to the injury [26]. We also found that continuous administration of angiotensin II to AM+/− mice induces severe glomerular injury with enhanced expression of TGF-β [47]. These results suggest that endogenous AM actively functions to protect the kidneys from injury.

Interestingly, RAMP2−/− phenotypes are embryonically lethal and similar to AM−/− phenotypes [25], [30], [48], [49], [50]. However, although both AM−/− and RAMP2−/− embryos exhibit abnormal vascular development, we detected no major renal deformities during embryogenesis. Recently, we developed a line of endothelial cell-specific RAMP2 KO (E-RAMP2−/−) mice. Most E-RAMP2−/− mice die perinatally; however, about 5% survive to adulthood. In the surviving E-RAMP2−/− adults, liver cirrhosis, cardiac fibrosis, and hydronephrosis all developed spontaneously. This organ damage is associated with marked fibrosis and oxidative stress and chronic inflammation. Thus, RAMP2 appears to be the critical determinant of AM function in adults, with AM-RAMP2 signaling exerting a protective effect by inhibiting inflammation and oxidative stress.

Glomerular lesions are the major pathological finding in diabetic nephropathy. From the results summarized above, we initially hypothesized that STZ-induced hyperglycemia would induce more severe glomerular lesions in RAMP2+/− than in WT kidneys. Both WT and RAMP2+/− showed similar blood glucose and BP levels, indicating that RAMP2 reduction did not have an effect on these parameters. We found, however, that RAMP2+/− mice administered STZ did not exhibit accelerated glomerular sclerosis when compared to WT mice. Instead, renal tubular injury due to ER stress was the major finding in the RAMP2+/− kidney, which suggests AM-RAMP2 exerts its protective effects by regulating the ER stress-UPR system.

The ER stress-UPR system is a stress response system initiated by accumulation of unfolded proteins [4]. Excessive accumulation of unfolded proteins disturbs cell homeostasis and can induce cell death [5]. It was recently reported, for example, that chronically elevated ER stress in pancreatic β cells induces diabetes [51], and that ER stress is increased in adipocytes in obese patients and is related to insulin resistance [52]. ER stress also appears to contribute to the pathogenesis of neurodegenerative diseases [53], [54]. In an experimental model, angiotensin II administration led to upregulated expression of BiP and CHOP and induction of cardiomyocyte apoptosis [55]. In the kidney, uremic toxins and urinary proteins reportedly cause injury by inducing ER stress [2], [3].

STZ injures pancreatic β cells, and the STZ administration model is often used to study hyperglycemia-induced organ damage. STZ-treated RAMP2+/− mice showed greater renal tubular injury than similarly treated WT mice. Notably, however, the tubular injury induced by STZ in RAMP2+/− kidneys was not mitigated by insulin treatment that normalized serum glucose levels. We therefore considered the renal tubular injury seen in RAMP2+/− kidneys to be a direct effect of STZ, not a secondary effect of hyperglycemia. Like other alkylating agents in the nitrosourea class, STZ is toxic to cells because it damages their DNA [56]. A glucose derivative, STZ is transported by GLUT2, which in addition to pancreatic β cells, is expressed in hepatocytes [57], small intestine epithelium [58], and renal tubules [59]. Our observation that autophagosome numbers are increased in tubule cells in STZ-treated RAMP2+/− kidneys was yet another observation highlighting the importance of the ER stress-UPR system in STZ-induced tubular injury.

In a set of *in vitro* experiments, we showed that STZ directly induces ER stress in cultured RPTECs, and that there is a corresponding upregulation of the activated form of the ER stress sensor, PERK. Interestingly, treating the cells with AM suppressed signaling downstream of PERK, in the eIF2α-ATF4-CHOP pathway, as well as CHOP-induced cell death. We then used TUN to confirm the tubular injury was caused by ER stress-induced cell death. TUN, which is commonly used to study the ER stress-UPR system, acts by inhibiting the synthesis of N-linked glycoproteins [39] and causes cell cycle arrest in G1 phase [36], which evokes ER stress. In the TUN-induced ER stress model, CHOP appeared to play a critical role by inducing cell death, which is consistent with the earlier report that CHOP KO mice are protected against TUN-induced renal tubular injury [37]. In RAMP2+/− mice, TUN administration caused tubule cell vacuolization and upregulation of the ER stress-related factors BiP and CHOP.

Our study using STZ and TUN may appear to be a rather specific situation. We chose these models because we found that these molecules clearly upregulate ER stress in renal tubular cells and RAMP2+/− are susceptible to them. Moreover, TUN is commonly used to evaluate ER stress-induced cellular and organ damage, as it directly evokes ER stress. However, drugs commonly used in clinical practice may also cause ER stress-related renal injury. Cisplatin is a major antineoplastic drug used in the treatment of solid tumors. However, it is also known to cause renal failure due to its preferential accumulation within the proximal tubule cells [42]. The mechanism of cisplatin-induced tubular injury is complex. Cisplatin causes a decrease in protein synthesis, as well as membrane peroxidation, mitochondrial dysfunction, and DNA injury. There is also evidence that ER stress is an important contributor to cisplatin-induced tubular damage [33]. We evaluated cisplatin-induced renal toxicity and found that RAMP2+/− kidneys showed greater tubular damage than WT kidneys. RAMP2+/− kidney showed upregulation of ER stress marker, as well as inflammatory and oxidative stress markers.

The specific signaling pathway, which directly upregulates UPR, is still largely unknown. UPR is evoked directly or indirectly through various stresses, including inflammation, toxin, oxidative stress, and other stresses. These stresses can cause the production of unfolded proteins, misfolded proteins, or simple overexpression of some proteins. As shown in the cisplatin study, inflammation and oxidative stress were enhanced in RAMP2+/−. We speculate that one explanation for the enhanced ER-stress in RAMP2+/− is the enhanced inflammation and oxidative stress, as endogenous AM has been shown to suppress excess inflammation and oxidative stress [13]. Another possibility is that RAMPs may work as chaperones, which carry some GPCRs from ER to the cell membrane [60]. RAMPs bind to other GPCRs including calcitonin receptor, parathyroid hormone receptor, and glucagon receptor, and not only AM receptor CLR, although their function with other GPCRs is largely unknown. In RAMP2+/−, reduction in the receptor trafficking of GPCRs might cause accumulation of excess GPCRs at ER, which might directly influence the enhanced ER stress.

In this study, we also analyzed whether exogenous AM treatment can protect against renal injury. In the TUN model, we found AM treatment can reduce the tubular injury in wild-type mice, however, it was not very effective in RAMP2+/− mice. This result again clearly indicates that the AM-RAMP2 system works to prevent organ damage. It has been reported that the blood AM level is elevated in patients with renal failure. Moreover, AM is elevated in proportion to the severity of the disease [22]. From the data obtained in this study, we speculate that elevation of AM in CKD functions as a compensatory mechanism against organ damage, although more intensive clinical studies are necessary in the future.

In this study, we did not find that hyperglycemia itself is involved in ER stress-evoked renal tubular injury, as the normalization of glucose levels in the STZ model could not rescue the injury. To clarify if the accumulation of metabolic abnormalities over a long period can also cause tubular injury, further studies in type II diabetic models or high fat-diet induced obese models should be conducted in the future.

Because elevated ER stress could be the cause of various diseases, chemicals that modulate ER stress are attracting attention due to their potential for clinical application. One of these compounds, 4-phenylbutyric acid (4-PBA), a non-peptide chaperone that can reduce ER stress by suppressing BiP and PERK [61], reportedly prevents the progression of diabetic nephropathy. In addition, it is anticipated that various endogenous molecules may eventually serve as new therapeutic targets. For example, nitric oxide (NO) reportedly reduces ER stress and protects against renal ischemia-reperfusion injury [62]. In the present study, we found that endogenous AM-RAMP2 signaling in renal tubule cells works to protect against renal injury by suppressing ER stress. Not surprisingly, the clinical application of AM is being much anticipated [63], [64], [65], [66], [67]. Unfortunately, AM is a peptide with a short half-life in the bloodstream, which limits its usefulness for the treatment of chronic diseases. However, we were able to mimic AM function by modulating RAMP2, which suggests RAMP2 could be a useful therapeutic alternative. Because RAMP2 is a low molecular weight protein, structural analysis and the synthesis of specific agonists or antagonists are much more realistic compared with the AM receptor CLR, which belongs to seven-transmembrane domain GPCRs. In this context, our findings provide a clear basis for the development of drugs to modulate RAMP2, and thereby, the renal effects of AM.

## Supporting Information

Figure S1 **Comparison of gene expression and ERK/pERK between wild-type and RAMP2+/− mice.** (A) Quantitative real-time PCR analysis of VE-cadherin, p53, and TGF-β gene expression in kidney. Data from the wild-type control group were assigned a value of 1. n = 5 in each group. Bars are means ± SEM. (B) Immunohistostaining of ERK and pERK in the section of kidney. Bars = 100 µm. No change was detected between wild-type and RAMP2+/− mice.
(EPS)

Figure S2 **Effect of STZ administration in other organs expressing GLUT2.** H&E staining of intestine, pancreas, and liver sections sampled from wild-type or RAMP2+/− with or without STZ administration. STZ administration caused cell death in these organs, however, the difference was not apparent between wild-type and RAMP2+/−. Bars = 50 µm.
(EPS)

Figure S3 **ER-stress related gene expression in STZ model.** Quantitative real-time PCR analysis of BiP and CHOP after 4 weeks with STZ (250 mg/kg) administration. Data from the wild-type was assigned a value of 1. n = 5 in each group. Bars are means ± SEM. *p<0.05.
(EPS)

Figure S4 **Urinary albumin level and megalin expression in STZ model.** (A) Comparison of urinary albumin after 4 weeks of STZ (250 mg/kg) administration. (B) Quantitative real-time PCR analysis of megalin with or without STZ. No change was detected between wild-type and RAMP2+/− mice.
(EPS)

Figure S5 **Proximal tubule changes in STZ administration.** (A) Toluidine blue staining of renal cortex obtained from each mouse after 2 weeks of STZ (250 mg/kg) administration. Bars = 50 µm. (B) Low magnification electron micrograph of a section of proximal tubule. In RAMP2+/−, tubular atrophy, microvesicular degeneration, and protrusion of luminal side of cell membrane were prominent.
(EPS)
